# Physical Exercise Mitigates Motor and Muscular Deficits in the 3xTg‐AD Model of Alzheimer's Disease

**DOI:** 10.1002/alz70860_097399

**Published:** 2025-12-23

**Authors:** José de Jesús Andrade, Sofia Díaz‐Cintra, Omar Emiliano Aparicio Trejo, Azucena Vázquez‐Aguilar, Ericka A De los Rios Arellano, Luis Oskar Soto‐Rojas

**Affiliations:** ^1^ Universidad Nacional Autónoma de México, Querertaro, QA, Mexico; ^2^ Instituto de Neurobiología UNAM, Queretaro, QA, Mexico; ^3^ Universidad Nacional Autonoma de México, Mexico city, EM, Mexico; ^4^ UNAM, Mexico, Mexico; ^5^ Universidad Nacional Autonoma de México, Queretaro, QA, Mexico; ^6^ Facultad de Estudios Superiores Iztacala, Universidad Nacional Autónoma de México, Carrera de Médico Cirujano, MEXICO CITY, EM, Mexico

## Abstract

**Background:**

Alzheimer's disease (AD) is the most common neurodegenerative disorder, marked by beta‐amyloid plaques and tau protein tangles, leading to cognitive decline and motor impairments in advanced stages. The motor symptoms that could appear are bradykinesia, rigidity, gait disturbances, and a reduction in muscle strength and mass, all of which significantly affect patients' quality of life and increase their dependency. The triple‐transgenic 3xTg‐AD mouse model, carrying three mutations linked to hereditary AD, is widely used for research. Physical exercise, a non‐pharmacological intervention, shows promise in managing AD symptoms, though its impact on motor and neuromuscular alterations remains unclear.

**Method:**

A total of 64 male mice, aged 12 months, were used and divided into four groups (*n* = 16 per group): 3xTg‐AD sedentary, 3xTg‐AD exercise, Non‐Tg sedentary, and Non‐Tg exercise. A 16‐week physical exercise intervention was conducted using a mixed protocol combining voluntary exercise (3 times a week) and forced exercise (2 times a week). At the end of the intervention, behavioral tests were performed to evaluate locomotor activity, gait, strength, coordination, and balance using the open field test, four‐limb pressure test, and the beam walk test, respectively. Additionally, memory was assessed using the novel object recognition test. Muscle mass was analyzed through bioimpedance studies and histological techniques. Finally, high‐resolution respirometry analysis was conducted at cerebral and muscular levels to examine mitochondrial activity.

**Result:**

Physical exercise improved motor impairments in the advanced stages of the AD model, positively impacted memory, reduced muscle atrophy, and promoted the enhancement of mitochondrial activity.

**Conclusion:**

The physical exercise intervention for AD patients is promising for reducing the effect of the disease on the locomotor system, preserving functionality, and preventing muscle and brain deterioration.

